# Segmental differences in upregulated apical potassium channels in mammalian colon during potassium adaptation

**DOI:** 10.1152/ajpgi.00181.2015

**Published:** 2016-09-08

**Authors:** Matthew D. Perry, Vazhaikkurichi M. Rajendran, Kenneth A. MacLennan, Geoffrey I. Sandle

**Affiliations:** ^1^Leeds Institute of Biomedical and Clinical Sciences, St James's University Hospital, Leeds, United Kingdom;; ^2^Department of Biochemistry, West Virginia University, Morgantown, West Virginia; and; ^3^Leeds Institute of Cancer and Pathology, St James's University Hospital, Leeds, United Kingdom

**Keywords:** BK channels, colonic K^+^ adaptation, rat colon

## Abstract

Rat proximal and distal colon are net K^+^ secretory and net K^+^ absorptive epithelia, respectively. Chronic dietary K^+^ loading increases net K^+^ secretion in the proximal colon and transforms net K^+^ absorption to net K^+^ secretion in the distal colon, but changes in apical K^+^ channel expression are unclear. We evaluated expression/activity of apical K^+^ (BK) channels in surface colonocytes in proximal and distal colon of control and K^+^-loaded animals using patch-clamp recording, immunohistochemistry, and Western blot analyses. In controls, BK channels were more abundant in surface colonocytes from K^+^ secretory proximal colon (39% of patches) than in those from K^+^-absorptive distal colon (12% of patches). Immunostaining demonstrated more pronounced BK channel α-subunit protein expression in surface cells and cells in the upper 25% of crypts in proximal colon, compared with distal colon. Dietary K^+^ loading had no clear-cut effects on the abundance, immunolocalization, or expression of BK channels in proximal colon. By contrast, in distal colon, K^+^ loading *1*) increased BK channel abundance in patches from 12 to 41%; *2*) increased density of immunostaining in surface cells, which extended along the upper 50% of crypts; and *3*) increased expression of BK channel α-subunit protein when assessed by Western blotting (*P* < 0.001). Thus apical BK channels are normally more abundant in K^+^ secretory proximal colon than in K^+^ absorptive distal colon, and apical BK channel expression in distal (but not proximal) colon is greatly stimulated as part of the enhanced K^+^ secretory response to dietary K^+^ loading.

## Abstract (precis)

*Mammalian colon is an important K^*+*^ homeostatic organ. In rats, chronic dietary K^*+*^ loading stimulates apical BK channel-mediated pan-colonic K^*+*^ secretion, the overall K^*+*^ secretory response being greater distally than proximally. Here we show that K^*+*^ loading induced a 3.5-fold increase in BK channel abundance and increased BK protein expression in surface and upper crypt cells in distal colon, but not in proximal colon, highlighting the importance of the distal colon in maintaining K^*+*^ homeostasis*.

## NEW & NOTEWORTHY

*Mammalian colon is an important K^*+*^ homeostatic organ. In rats, chronic dietary K^*+*^ loading stimulates apical BK channel-mediated pan-colonic K^*+*^ secretion, the overall K^*+*^ secretory response being greater distally than proximally. Here we show that K^*+*^ loading induced a 3.5-fold increase in BK channel abundance and increased BK protein expression in surface and upper crypt cells in distal colon, but not in proximal colon, highlighting the importance of the distal colon in maintaining K^*+*^ homeostasis*.

mammalian colon is a potassium (K^+^) secretory organ. Its capacity for K^+^ secretion increases during chronic dietary K^+^ loading and chronic renal insufficiency, as a result of adaptive changes in K^+^ transport processes within colonic epithelial cells (7, 19, 25, 31, 36). The mechanisms underlying colonic K^+^ adaptation have been studied extensively in rat colon, which normally exhibits distinct segmental differences in net K^+^ transport. Thus, under in vitro conditions, net K^+^ secretion is a feature of the proximal colon (6), whereas net K^+^ absorption occurs in the distal colon as a result of K^+^-H^+^-ATPase-mediated apical K^+^ uptake and a negligible apical membrane K^+^ conductance (6, 17, 35). Chronic dietary K^+^ loading stimulates an active K^+^ secretory process throughout rat colon, enhancing net K^+^ secretion in the proximal segment and reversing net K^+^ absorption to net K^+^ secretion in the distal segment (8, 9). Enhancement of net K^+^ secretion in the proximal segment reflects a twofold increase in the serosa-to-mucosa K^+^ flux without a change in the mucosa-to-serosa K^+^ flux (8), whereas in the distal segment stimulation of net K^+^ secretion reflects a threefold increase in the serosa-to-mucosa K^+^ flux, together with a 66% decrease in the mucosa-to-serosa K^+^ flux (9). The cellular changes underlying these changes in unidirectional K^+^ fluxes include amplification of the basolateral membrane area, leading to enhanced cellular K^+^ uptake via increased Na^+^-K^+^-ATPase activity (9, 15, 19); a rise in intracellular K^+^ concentration, thereby increasing the chemical gradient favoring apical K^+^ exit into the lumen (31); and enhancement of the tetraethylammonium (TEA) chloride-inhibitable apical conductance (presumed to be an apical K^+^ conductance) (31).

Patch-clamp studies have identified high-conductance apical K^+^ (BK) channels at relatively low abundance in surface cells in the distal colon of rats fed a control diet, which increased substantially during chronic dietary K^+^ loading (4), but it unclear whether a similar change occurs in rat proximal colon. It is possible that a pan-colonic increase in apical K^+^ conductance is part of the adaptive response to dietary K^+^ loading, but it is also unclear whether this reflects increased apical BK channel protein expression, or an increase in BK channel activity, or a combination of both possibilities. There is now compelling evidence from studies in mouse distal colon that aldosterone plays a critical role in upregulating apical BK channels as part of the colonic K^+^-adaptive response to dietary K^+^ loading (42). A high-K^+^ diet for 4 days (which increased plasma aldosterone levels) stimulated luminal iberiotoxin-sensitive electrogenic K^+^ secretion and twofold increases in BK channel α- and β-subunit mRNAs in normal mice, while in vitro addition of aldosterone stimulated a similar increase in electrogenic colonic K^+^ secretion in normal mice that was prevented by spirololactone, a mineralocorticoid receptor antagonist. By contrast, the high-K^+^ diet had no effect on distal colonic K^+^ secretion in BK α-subunit-deficient mice, indicating that enhanced colonic K^+^ secretion was mediated via aldosterone-dependent apical BK channels (42).

Aldosterone appears to also regulate colonic BK channel activity by increasing intracellular pH. Hyperaldosteronism secondary to chronic dietary K^+^ loading promotes intracellular alkalinization as well as stimulating apical BK channel activity, both changes being reversed by the Na^+^-H^+^ exchange inhibitor ethylisopropyl amiloride (30). Thus, in addition to the aldosterone-dependent increases in apical BK channel expression that occur in mouse and rat distal colon during chronic dietary K^+^ loading, intracellular alkalinization secondary to aldosterone-stimulated Na^+^-H^+^ exchange may activate newly synthesized BK channels as well as “latent” BK channels already present within the apical membrane. The aim of the present study was to explore the role of apical BK channels in the enhanced K^+^ secretory responses of rat proximal and distal colon to chronic dietary K^+^ loading. We found that while the biophysical properties of BK channels were similar in rat proximal and distal colon, and were unchanged by K^+^ loading, this dietary perturbation increased the abundance of BK channels (assessed by patch-clamp analysis) and increased the expression of BK channels along the upper crypts (assessed by immunolocalization and Western blot analysis) in distal colon, whereas these changes were not seen in the proximal colon.

## METHODS AND MATERIALS

### 

#### Preparation of animals.

Male Wistar rats weighing 200–250 g were fed either standard rat chow (20 g per day; 0.2 mmol K^+^/g; “control” animals) or a paste chow diet supplemented with KCl (20 g per day; 1.6 mmol K^+^/g; “K^+^-loaded” animals) for 10–14 days and allowed access to tap water ad libitum. This method of chronic dietary K^+^ enrichment has previously been used to induce all of the changes in cellular K^+^ transport required for enhanced active K^+^ secretion in rat colon (7). Animals were killed by inducing CO_2_ narcosis prior to dislocation of the neck. These animal procedures were approved by the UK Home Office. Segments of colon (4 cm) were removed from just adjacent to the cecum (proximal) or from just above the pelvic brim (distal).

#### Isolation of surface colonocytes.

Single and small clumps of surface colonocytes were isolated by an adaptation of a Ca^2+^ chelation technique previously described (43). Colonic segments were flushed three times with 10 ml of an ice-cold solution containing (in mmol/l) 154 NaCl, 10 glucose, and 0.5 dithiothreitol; opened longitudinally to expose the mucosal surface; and then incubated (30 min at room temperature) in 30 ml of a solution containing (in mmol/l) 30 NaCl, 5 Na_2_EDTA, 8 HEPES, and 0.5 dithiothreitol, buffered to pH 7.6 with 1 mol/l Trizma base. During incubation, surface colonocytes were released by gentle shaking at 10-min intervals, isolated by centrifugation (600 *g* for 5 min), and resuspended for 5 min in 25 ml of a high-K^+^ solution containing (in mmol/l) KCl 135, CaCl_2_ 1.2, MgCl_2_ 1.2, Na^+^ butyrate 5, glucose 5, and HEPES 10, buffered to pH 7.4 with 1 mol/l KOH, and supplemented with 1 mg/ml collagenase Type 1A. Cells were recentrifuged and resuspended in 20 ml of the high-K^+^ solution, and the protocol was repeated three times before finally resuspending the cells in 5 ml of the high-K^+^ solution kept on ice. After the release of surface colonocytes, histology of the residual mucosal sheets confirmed that Ca^2+^ chelation removed surface cells and occasionally cells in the upper 25% of the crypts (data not shown), indicating that the isolate consisted mainly of surface colonocytes.

#### Patch-clamp recording.

Single-channel recordings were obtained in cell-attached and excised inside-out configurations from the cell membrane of isolated surface colonocytes. Although these cells were nonpolarized, previous patch-clamp studies showed that dietary K^+^ loading resulted in similar increases in the abundance of “apical” BK channels in rat distal colon, irrespective of whether recordings were obtained from the apical membrane of surface colonocytes around the luminal openings of intact isolated crypts or the cell membrane of single surface colonocytes (4). It therefore seems likely that the cell membrane of isolated surface colonocytes is dominated by BK channels originating from the apical pole of the cell (see discussion).

Patch pipettes were prepared from fiber-filled borosilicate capillary tubing (OD 1.5 mm, ID 0.86 mm; Harvard Apparatus, Edenbridge, UK) and fire polished to give pipette and membrane seal resistances of 5–10 and 10–15 MΩ, respectively. The bath solution contained (in mmol/l) 140 NaCl, 4.5 KCl, 1.2 CaCl_2_, 1.2 MgCl_2_, 5 glucose, 5 Na^+^ butyrate, and 10 HEPES, buffered to pH 7.4 with 1 mol/l NaOH. The pipette solution contained (in mmol/l) 145 KCl, 1.2 CaCl_2_, 1.2 MgCl_2_, and 10 HEPES, buffered to pH 7.4 with 1 mol/l KOH. Experiments were done at 20–22°C rather than at 37°C to maintain viability (44). Membrane patches were clamped at voltages referenced to the pipette interior via the patch-clamp amplifier (List Electronics model EPC-7, Darmstadt, Germany). Currents were stored on videotape after pulse code modulation (Sony model PCM 701ES, Tokyo, Japan) and later were filtered (600 Hz, −3 dB, four-pole Butterworth response filter) and loaded (sampling frequency 4 kHz) into computer memory via a Labmaster TL1 interface and TM40 A/D converter (Axon Instruments, Foster City, CA). Data were analyzed with pClamp software version 5.7 (Axon Instruments) and a program written in Quick Basic 4.0 (Microsoft) to determine single-channel open probability (P_O_), calculated as P_O_ = (∑*nt*_*n*_)/*N*, where *N* is the maximum number of channels seen to be open simultaneously during the recordings, *n* is the state of the channels (0, closed; 1, one channel open, etc.), and *t*_*n*_ is the time spent in state *n*.

#### Immunohistochemical studies.

Segments of proximal and distal colon from control and K^+^-loaded animals (1 cm^2^) were fixed in 10% formaldehyde in phosphate-buffered saline for 24–48 h, dehydrated with increasing concentrations (50–100%) of ethanol, and embedded in paraffin wax. Sections of 3 μm were transferred to electrostatically charged superfrost slides (BDH) and fixed by warming the slides to 60–65°C. Sections were dewaxed with warm (37°C) xylene, rehydrated by exposure to decreasing concentrations (100-50%) of ethanol, and finally rinsed several times with water. Endogenous peroxidase activity was blocked by incubating with 3% H_2_O_2_ (diluted with deionized water) at room temperature for 10 min, followed by rinsing with tap water for 5 min. Sections were heated in a microwave oven at full power for 13 min (ensuring boiling for exactly 10 min), then cooled on ice for 10 min and rinsed with running water. Nonspecific binding was blocked by incubating sections for 30 min in normal swine serum diluted 1:10 with 50 mmol/l Tris-buffered saline (TBS) containing 0.1% (wt/vol) Na^+^ azide, then washing with TBS for 5 min. The primary antibody (anti-BK) was a polyclonal antibody raised in rabbit against highly purified fusion protein of *Schistosoma japonicum* glutathione *S*-transferase and the COOH-terminal end (residues 1098–1196) of the mouse *Slowpoke* (*mSlo*) channel α-subunit (Alomone Labs, Jerusalem, Israel). This anti-BK antibody detects a protein of the predicted size (∼125 kDa) in Western blots in rabbit (46) and mouse (1) tissue, and its specificity has been confirmed in mouse tissue by preadsorption of the BK (amino acids 1098–1196) antibody with the antigen peptide, which abolished the positive control and the bands at 125 kDa (1). The antibody was applied at a dilution of 1:50 for 60 min and removed by twice washing with TBS. The secondary antibody was biotinylated goat anti-rabbit immunoglobulin (Dako, High Wycombe, UK) applied at a dilution of 1:400 for 30 min and removed by two washes with TBS. Labeling was completed by adding streptavidin/horseradish peroxidase avidin-biotin complex for 30 min, which was then removed by washing twice with TBS. Sections were then incubated with diaminobenzidine solution for 15 min and rinsed with tap water for 5–15 min. Hematoxylin counterstain was applied for 30 s and removed by washing with running water for 2 min.

#### Western blot analyses.

Brain and mucosal scrapings of segments of proximal and distal colon from control and K^+^-loaded animals were suspended in 20 ml of ice-cold lysis solution containing 50 mmol/l Tris buffered to pH 8.0, 0.5% SDS, 1 mmol/l phenylmethylsulfonylfluoride, 4 μg/ml pepstatin A, and one tablet of complete protease inhibitor/50 ml solution (Roche Applied Science, Indianapolis, IN). Homogenized tissues were centrifuged (2,000 *g* for 15 min) to remove cell debris, and 16-μl aliquots of the supernatants were mixed with an equal volume of Laemmli buffer, then heated for 5 min at 95°C. Concentrations of extracted protein were determined by a modification of the Lowry technique (27) and 10-μl samples (30 μg protein) were subjected to SDS-polyacrylamide gel electrophoresis. Gels were transferred onto nitrocellulose membranes (Hybond ECL, Amersham Pharmacia Biotech, Piscataway, NJ) and blocked overnight in TBST (Tris-buffered saline, 0.1% Tween 20) containing 5% fat-free dried milk at 4°C. Blots were then incubated for 2 h at room temperature with blocking buffer containing anti-BKα primary antibody (Alomone Labs) at 1:50 dilution, or anti-GAPDH antibody (Santa Cruz Biotechnology, Hercules, CA) at 1:2,500 dilution, following which they were washed with TBST containing 5% fat-free dried milk, incubated for 1 h at room temperature in blocking buffer containing horseradish peroxidase-conjugated goat anti-rabbit IgG (Jackson ImmunoResearch Laboratories, West Grove, PA) at 1:5,000 dilution, and again washed with TBST. Immune complexes were detected on film by enhanced chemiluminescence (Amersham Pharmacia Biotech). Arbitrary units of BKα proteins normalized to GAPDH were quantitated with Personal Densitometer SI (Molecular Dynamics).

#### Statistics.

Numerical data are shown as mean values ± SE. In most cases, Student's *t*-test (unpaired) was used to compare differences in the mean values between similar sited colonic segments from control and K^+^-loaded animals, *P* < 0.05 (two-tailed) being taken to indicate statistical significance. The Mann-Whitney *U*-test was used to compare BK channel frequency distribution between cell-attached patches on surface colonocytes from the two groups of animals. The data were further modeled as a Poisson distribution (correcting for overdispersion by scaling errors using the ratio of residual deviance/degrees of freedom) to estimate changes in K^+^ channel abundance in response to dietary K^+^ loading.

## RESULTS

### 

#### Effect of dietary K^+^ loading on BK channel abundance.

**Fig. 1.**
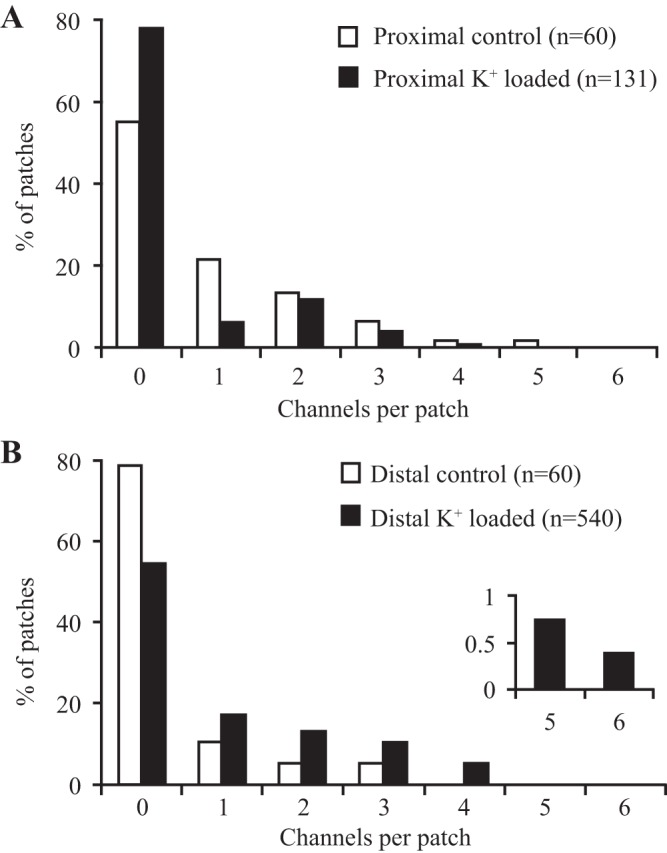
Effect of dietary K^+^ loading on the frequency distribution of BK channels in surface cells isolated from rat proximal (*A*) and distal (*B*) colon. The number of BK channels in cell-attached patches was decreased by K^+^ loading in proximal colon, while the opposite effect was seen in distal colon. *Inset* in *B* shows that a small number of patches in cells from distal colon of K^+^-loaded animals contained up to 6 BK channels.

Table 1 shows that, in the proximal colon, the abundance of cell-attached patches containing apical BK channels in surface colonocytes in dietary K^+^-loaded animals (26%) was less than in controls (39%). This corresponded to a decrease in the frequency distribution of BK channels within each patch (Fig. 1; *P* < 0.05, Mann-Whitney *U*-test). Thus 23.3% of cell-attached patches contained two or more BK channels in controls, compared with 16% of cell-attached patches in dietary K^+^-loaded rats, which represents a 0.52-fold (95% confidence limits, 0.36 and 0.76) decrease in K^+^ channel density after remodeling the data as a Poisson distribution.

**Table 1.** Abundance of BK channels in surface colonocytes isolated from proximal and distal colon of control and dietary K^+^-loaded animals

Colonic Segment	Number of Patch Attempts	Number of Patches (% Success)	Number of Patches with BK Channels (% Patches with BK Channels)
Proximal (control)	401	54 (13%)	21 (39%)
Distal (control)	273	33 (12%)	4 (12%)
Proximal (K^+^ loaded)	1,114	116 (10%)	30 (26%)
Distal (K^+^ loaded)	6,494	1081 (17%)	440 (41%)

Table 1 also shows that in distal colon the abundance of cell-attached patches containing apical BK channels in surface colonocytes from dietary K^+^-loaded animals (41%) was greater than in controls (12%), as reported previously (4). Dietary K^+^ loading also increased the frequency distribution of BK channels within each patch when compared with controls (Fig. 1; *P* < 0.02, Mann-Whitney *U*-test). Thus only 5.3% of cell-attached patches on surface colonocytes isolated from controls contained two or more BK channels, in contrast to 29% in dietary K^+^-loaded animals, which represents a 2.65-fold (95% confidence limits, 1.26 and 5.58) increase in K^+^ channel density after remodeling the data as a Poisson distribution. These results confirm earlier observations that dietary K^+^ loading increases the level of spontaneous BK channel activity in rat distal colon (4).

#### Properties of BK channels in proximal and distal colon.

**Fig. 2.**
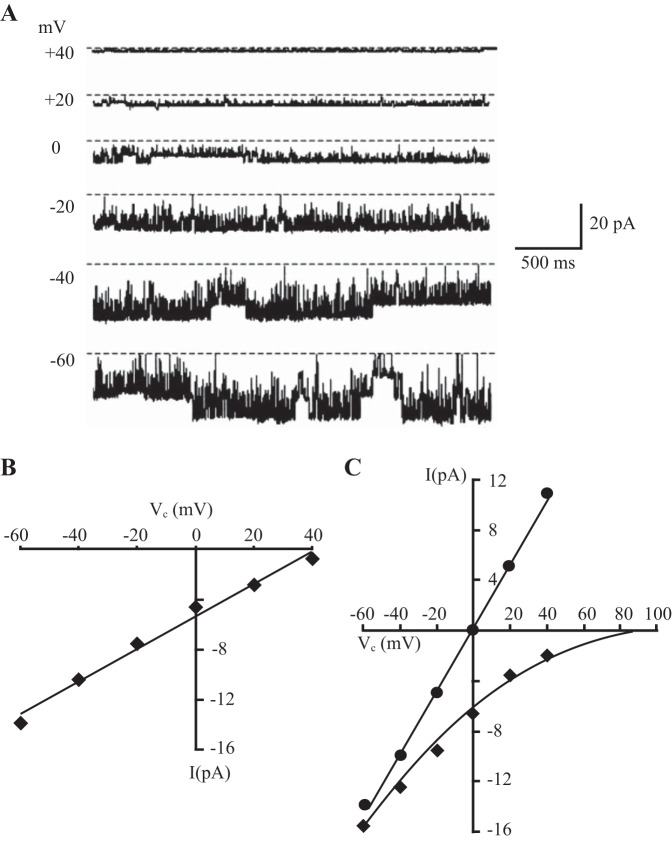
Characteristics of BK channels in proximal colon of dietary K^+^-loaded animals. *A*: typical current recordings from a cell-attached patch showing 3 BK channels at different command potentials referenced to the pipette interior (NaCl solution in bath and KCl solution in pipette). Dashed lines indicate 0 current levels. Downward current deflections indicate K^+^ flow from the pipette to the cell. *B*: current (*I*)-voltage (V_c_) relationship of the recordings in *A*, from which the single-channel conductance (135 pS) was calculated by linear regression analysis. *C*: K^+^ selectivity of the BK channel, determined from the current (*I*)-voltage (V_c_) relationship while recording from an excised inside-out patch, first with NaCl solution in the bath and KCl solution in the pipette (⧫; data fitted to the current form of the Goldman-Hodgkin-Katz equation), which indicated a channel K^+^-to-Na^+^ selectivity ratio of 254 ± 11:1 (*n* = 7); then with KCl solution in the bath and the pipette (●; data fitted by linear regression analysis), indicating a single-channel conductance of 210 ± 12 pS. *I*, single-channel current; V_c_, command voltage.

BK channel characteristics in cell-attached patches on proximal and distal colonic surface cells were similar in control and dietary K^+^-loaded animals, and typical current recordings from a proximal colonic surface cell in a dietary K^+^-loaded animal are shown in Fig. 2*A*. In proximal colon, pooled cell-attached data from controls (*n* = 3) and K^+^-loaded animals (*n* = 11) indicated slope conductances and reversal potentials of 150 ± 5 pS and 42 ± 8 mV and 136 ± 5 pS and 38 ± 2 mV, respectively (Fig. 2*B*). Spontaneous BK channel activity (V_com_ = 0 mV) was high in both control (P_O_ 0.65 ± 0.16) and K^+^-loaded animals (P_O_ 0.77 ± 0.06) and was voltage independent under these conditions (NaCl solution in bath, KCl solution in pipette). Current-voltage relationships from seven excised inside-out patches (K^+^-loaded animals) indicated a reversal potential and P_K_/P_Na_ ratio of 85.4 ± 0.1 mV and 254:1, respectively (Fig. 2*C*). Changing the bath solution to high KCl produced a linear current-voltage relationship and a single-channel slope conductance of 210 ± 12 pS (Fig. 2*C*).

Similar data (not shown) were obtained in distal colonic surface cells. Pooled cell-attached data from controls (*n* = 2) and dietary K^+^-loaded animals (*n* = 13) indicated slope conductances and reversal potentials of 150 pS and 35 mV, and 150 ± 5 pS and 41 ± 2 mV, respectively. Spontaneous BK channel activity was again high in both control (P_O_ 0.75 ± 0.2) and K^+^-loaded animals (P_O_ 0.77 ± 0.06) and voltage independent under these conditions (NaCl solution in bath, KCl solution in pipette), where a high concentration of Ca^2+^ was present on both sides of the excised patches. Current-voltage relationships from three excised inside-out patches (K^+^-loaded animals) indicated a reversal potential and P_K_/P_Na_ ratio of 85.1 ± 0.1 mV and 269:1, respectively. Changing the bath solution to high KCl produced a linear current-voltage relationship and a single-channel slope conductance of 215 ± 6 pS. Taken together, these data indicate that the basic characteristics of BK channels in rat proximal and distal colon are similar and remain unchanged during chronic dietary K^+^ loading. Although we did not study the effects of specific BK channel inhibitors in this study, we have previously shown that apically applied 100 nM iberiotoxin completely inhibits cAMP-induced K^+^ secretion in rat distal colon by preventing the apical BK channel-mediated increase in unidirectional K^+^ flux from serosa to mucosa (38), and 100 nM penitrem A inhibits apical BK channels in human colon (21).

#### K^+^ loading and BK channel protein distribution and expression.

**Fig. 3.**
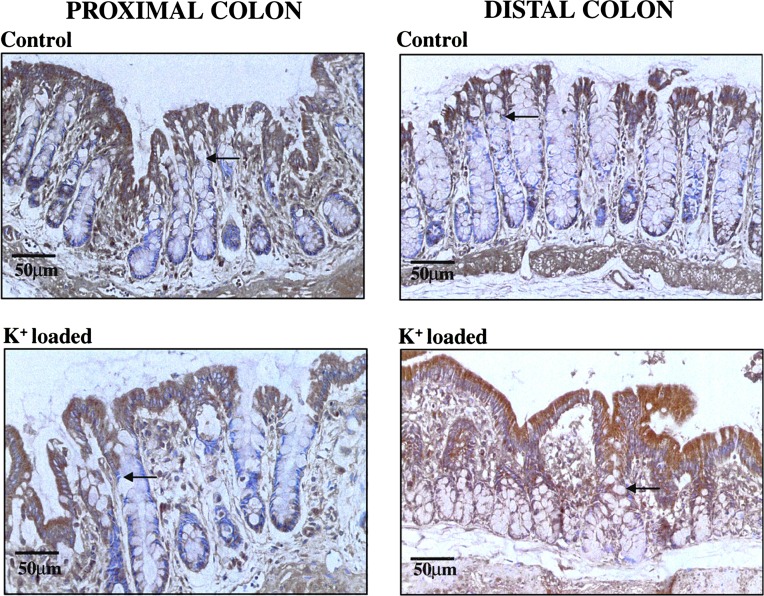
Effect of dietary K^+^ loading on the distribution of BK channels in rat proximal and distal colon. Antibody to BK channel α-subunit protein localized to surface cells and cells in the upper 25% of crypts (indicated by arrows) from proximal and distal colon of 3 control animals. Dietary K^+^ loading (3 animals) had no effect on the expression or distribution of BK channel α-subunit protein in the proximal colon but increased surface cell expression and cryptal distribution in the distal colon (indicated by arrows). Sections were counterstained with hematoxylin.

Immunostaining was performed using a primary antibody with proven specificity for the BK channel α-subunit protein (1). In proximal colon from controls, BK channel α-subunit protein localized to surface cells and cells in the upper 25% of the crypt axis, and the level of expression was unchanged by dietary K^+^ loading (Fig. 3). In controls, the distribution of BK channel α-subunit protein was similar in proximal and distal colon (Fig. 3), but the density of staining was lower in the K^+^ absorptive distal colon than in the K^+^ secretory proximal colon. However, unlike the proximal colon, K^+^ loading greatly enhanced the expression of BK channel α-subunit protein in surface colonocytes of the distal colon and also extended the distribution of channels along the upper 50% of the crypts (Fig. 3), in keeping with the increased abundance of apical BK channels demonstrated in the present and previous patch-clamp studies (4). Interestingly, BK channels are also expressed predominantly in surface colonocytes in rabbit and human distal colon (16, 25).

**Fig. 4.**
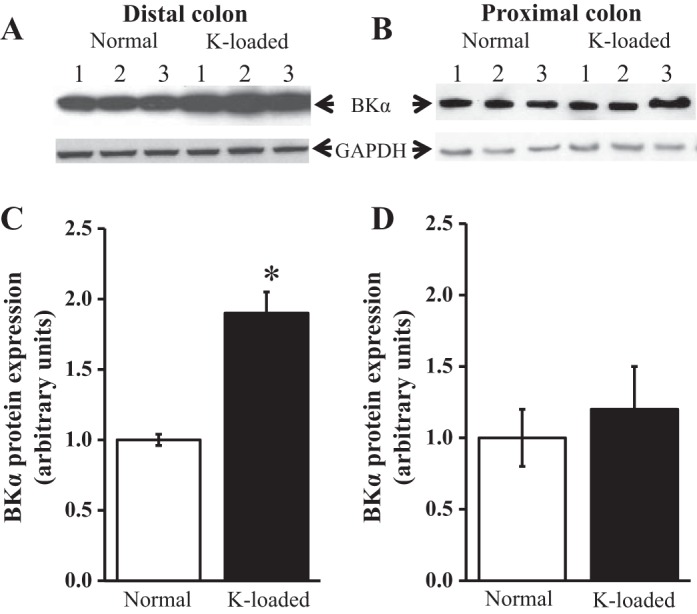
Effect of dietary K^+^ loading on BK channel expression in rat proximal and distal colon. *A*: Western blots of protein (30 μg per lane) from 3 control distal colons (*lanes 1*-*3*) and 3 K^+^-loaded distal colons (*lanes 4*-*6*). *B*: blots from 3 control proximal colons (*lanes 1*-*3*) and 3 K^+^-loaded proximal colons (*lanes 4*-*6*). Blots were repeated in 3 additional control and K^+^-loaded animals, so that paired proximal and distal colonic segments were obtained from 6 control and 6 K^+^-loaded animals. After probing with BKα antibody, blots were stripped and probed with anti-GAPDH antibody, GAPDH acting as internal control. Molecular sizes of BKα and GAPDH were ∼120 and 40 kDa, respectively. *C* and *D*: summaries of quantified protein band densities from 6 distal colons and 6 proximal colons, respectively. *P < 0.001.

Western blot analyses detected BK channel α-subunit protein in the proximal and distal colon of six control and six K^+^-loaded animals, the levels of which were normalized to the levels of GAPDH (Fig. 4). In the distal colon, levels of BK channel α-subunit protein were significantly greater (*P* < 0.001) in K^+^-loaded animals than in controls (Fig. 4, *A* and *C*), whereas in the proximal colon, K^+^ loading had no effect on the levels of BK channel α-subunit protein (Fig. 4, *B* and *D*), in keeping with the immunostaining data. The primary antibody also detected BK channel α-subunit protein in control rat brain, the level of which was unchanged by K^+^ loading.

## DISCUSSION

### 

#### Characteristics of BK channels in normal and K^+^-loaded proximal and distal colon.

In this study we show for the first time that apical BK channels in surface epithelial cells isolated from proximal and distal colon of rats fed a normal diet possess similar biophysical properties, which were unchanged during colonic K^+^ adaptation induced by chronic dietary K^+^ loading. Single-channel conductance in excised inside-out patches bathed in symmetrical KCl solution was ∼210 pS, similar to that reported in surface epithelial cells from human distal colon (4) and rat brain (13). These channels are highly selective for K^+^ over Na^+^, have a reversal potential close to the theoretical K^+^ reversal potential (E_K_), and are Ba^2+^ sensitive (4). We also found BK channel activity to be voltage independent, which most likely reflected the high concentration of Ca^2+^ (1.2 mM) present on both sides of the excised patches, since previous studies have shown BK channel activity in rat colon to be both voltage and Ca^2+^ sensitive, voltage sensitivity being apparent only at low “cytosolic” Ca^2+^ concentrations (4). The molecular structures of apical BK channels in proximal colon may be identical to those in distal colon, but it is possible that alternative splicing of RNA encoding the BK channel α-subunit, or segmental differences in the associated β-subunits or other regulatory proteins, may influence the effects of Ca^2+^, voltage, kinases, and other second messengers on channel gating. We recorded BK channel activity from isolated and presumably nonpolarized surface colonocytes, raising the possibility that these channels were derived from the basolateral rather than the apical membrane. However, we previously demonstrated mainly single BK channels in 9 of 90 (10%) cell-attached patches in isolated surface cells from distal colon of control animals, with multiple BK channels in 247 of 437 (57%) patches in isolated surface cells from distal colon of K^+^-loaded animals (4). The fact that almost identical observations were made during apical membrane recordings from surface cells in situ around the openings of isolated intact crypts [one BK channel seen in 1 of 11 (9%) patches in control animals, and up to five channels/patch seen in 9 of 13 (69%) patches in K^+^-loaded animals] led us to conclude that these BK channels originated from the apical membrane (4). Our finding that apical BK channels are localized mainly in surface epithelial cells in rat colon agree with previous studies in rabbit and human distal colon (16, 25), although apical BK channel expression appears to be largely restricted to colonic crypt cells in mice (40). It should be noted that the unitary conductance and kinetics of the apically derived BK channels we describe in the present and previous studies in rat and human colon (4, 30, 37) are fundamentally different from those of the infrequent 187-pS basolateral K^+^ channel with no known function identified in basal cells in rat distal colonic crypts (3) and the briefly opening 138-pS basolateral K^+^ channel previously identified in human colonic crypts (23, 39).

#### Segmental differences in colonic BK channel expression and abundance induced by K^+^ loading.

Based on immunostaining (Fig. 3) and Western blotting (Fig. 4) using antibody to BK channel α-subunit protein, we also show for the first time that chronic dietary K^+^ loading in rats markedly increases the expression of apical BK channels in the distal colon, but not in the proximal colon, despite previous reports that K^+^ loading stimulates net K^+^ secretion in both colonic segments (8, 9). Furthermore, patch-clamp experiments revealed that BK channel abundance was also significantly greater in distal colon from K^+^-loaded animals than controls, whereas channel abundance tended to be lower in proximal colon from K^+^-loaded animals than controls (Table 1), although this difference in the proximal colon may not be physiologically significant. Nevertheless, previous studies in rat proximal colon showed that dietary K^+^ loading doubled the unidirectional K^+^ flux from serosa to mucosa (from 0.58 ± 0.05 to 1.26 ± 0.20 μeq·h^−1^·cm^2^) without changing the unidirectional K^+^ flux from mucosa to serosa, resulting in a significant increase in net K^+^ secretion from −0.23 ± 0.05 to −0.79 ± 0.17 μeq·h^−1^·cm^2^ (8). Assuming that apical BK channels contribute to this proximal colonic K^+^ secretory response, it follows that, in the absence of increased BK channel expression/abundance, an additional factor may stimulate the activity of preexisting channels. It is therefore of interest that apical BK channels in surface cells from rat distal colon are pH sensitive, and the increases in apical BK channel activity and intracellular alkalinization (most likely reflecting increased aldosterone-dependent Na^+^-H^+^ exchange) seen in distal colon of K^+^-loaded animals are both reversed by the Na^+^-H^+^ exchange inhibitor ethylisopropyl amiloride (30). Thus it is conceivable that intracellular alkalinization during chronic K^+^ loading stimulates apical BK channel activity in the proximal colon (where BK channel protein expression is unchanged), as well as in the distal colon (where BK channel protein expression is increased). In this regard, it should be emphasized that both an increase in the dietary K^+^ load and a significant degree of secondary hyperaldosteronism are required to transform rat distal colon from being a normally net K^+^-absorptive epithelium into a net K^+^-secretory epithelium during chronic dietary K^+^ loading (7). The increase in dietary K^+^ load per se may be sufficient to enhance apical BK channel expression in distal colonic surface cells, since dietary K^+^ enrichment increases both the macroscopic K^+^ conductance and K^+^ currents flowing across the apical membrane of renal cortical collecting duct cells in adrenalectomized rabbits (26). In addition to the increase in apical BK channel expression/activity, enhanced colonic K^+^ secretion induced by chronic dietary K^+^ loading is associated with amplification of the basolateral membrane area and Na^+^-K^+^-ATPase activity in colonic epithelial cells (19), both of which enhance basolateral K^+^ uptake and contribute to the increase in the unidirectional K^+^ flux from serosa-to-mucosa. Basolateral membrane amplification may also increase basolateral Na^+^-K^+^-2Cl^−^ activity, which contributes to enhanced basolateral K^+^ uptake in colonic K^+^ hypersecretory states, since serosal bumetanide markedly inhibited the unidirectional serosa-to-mucosa K^+^ flux and abolished net K^+^ secretion in aldosterone-stimulated K^+^ secretory rat distal colon (45), and also abolished adrenaline-stimulated electrogenic K^+^ secretion in guinea pig distal colon (29).

Although chronic dietary K^+^ loading stimulates active K^+^ secretion in rat proximal colon (8), the prevalence of apical BK channels in proximal colon appeared to be lower in K^+^-loaded animals (26%) than in controls (39%). This apparent difference may not be physiologically significant, since neither immunostaining (Fig. 3) nor Western blotting (Fig. 4) detected decreases in apical BK channel protein expression in this colonic segment during K^+^ loading. It should be noted that Ca^2+^-sensitive, intermediate conductance, and clotrimazole-inhibitable K^+^ (IK_Ca_) channels contribute to the apical membrane K^+^ conductance in rat proximal colon, which mediate K^+^ secretion stimulated by thapsigargin and carbachol (18). However, Western blot analyses indicated that K^+^ loading had no effect on IK_Ca_ channel protein expression, either in the proximal or the distal colonic segments (data not shown).

By contrast, in the distal colon, in which chronic dietary K^+^ loading stimulates an even greater K^+^ secretory response than in proximal colon (14), the prevalence of apical BK channels increased 3.5-fold (from 12% in controls to 41% in K^+^-loaded animals), together with a 2.65-fold increase in the frequency distribution of the channels. This difference is in broad agreement with previous single-channel studies using isolated surface cells from rat distal colon (4). This patch-clamp evaluation is supported by the immunostaining studies, which indicated that apical BK channel protein expression was enhanced in the distal colon by K^+^ loading, and channel protein was distributed more widely along the surface cell-crypt cell axis in K^+^-loaded animals (upper 50% of crypts) than in controls (upper 25% of crypts). Furthermore, Western blot analyses demonstrated that the increase in apical BK channel prevalence observed in the distal colon in response to K^+^ loading reflected an increase in BK channel protein expression. Thus the increase in apical K^+^ conductance that occurs in rat distal colon during dietary K^+^ loading reflects increased apical BK channel synthesis and membrane insertion, as well as the stimulatory effect of Na^+^-H^+^ exchange-mediated cytosolic alkalinization on the activity of these new channels and any latent BK channels already present in the membrane (30). This view is consistent with data from site-directed intracellular microelectrode and conductance scanning studies, which demonstrated that the negligible macroscopic apical K^+^ conductance of control rat distal colon was significantly enhanced by aldosterone, most of the change occurring in surface cells and to a lesser extent in cells located in the upper half of the crypts (11, 22). However, it should be noted that while BK channel upregulation in response to K^+^ loading occurs in surface cells and upper crypt cells in rat distal colon, dietary K^+^ loading in mice upregulates apical BK channels along the length of distal colonic crypts (42), whereas BK channel expression is much lower in surface colonocytes (40). Thus there appear to be significant species differences in colonic apical BK channel expression, since apical BK channels are present mainly in the surface cells of rabbit distal colon (12), and immunostaining demonstrated increased expression of apical BK channels in surface colonocytes and upper crypt cells in patients with end-stage renal disease who exhibited increased distal colonic K^+^ secretion (25).

#### Relationship between electrogenic K^+^ secretion and electrogenic Na^+^ absorption in distal colon during K^+^ loading.

The high single-channel conductance and activity of apical BK channels strongly suggests that they play an important role in mediating increased K^+^ secretion induced by chronic dietary K^+^ loading. We observed a uniformly high level of voltage-independent BK channel activity in proximal and distal colon of control and K^+^-loaded animals, but we have previously shown that channel activity increased at depolarizing voltages using “intracellular” Ca^2+^ concentrations to bathe the cytosolic side of excised inside-out patches (4). Both chronic dietary K^+^ loading and chronic dietary Na^+^ depletion lead to secondary hyperaldosteronism and stimulate electrogenic Na^+^ absorption (as well as electrogenic K^+^ secretion) in rat distal colon (2, 6, 31, 34). However, it should be emphasized that, in rat distal colon, dietary K^+^ loading stimulates amiloride-sensitive electrogenic Na^+^ absorption without changing electroneutral NaCl absorption, which is the sole mechanism of Na^+^ absorption in this colonic segment in control animals (2). Chronic dietary Na^+^ depletion, which elicits at least 10-fold higher levels of plasma aldosterone than chronic dietary K^+^ loading (24), stimulates an even greater level of electrogenic Na^+^ absorption and inhibits electroneutral NaCl absorption in rat distal colon (10), while in rat proximal colon (where basal Na^+^ absorption reflects electroneutral Na^+^-H^+^ exchange), Na^+^ depletion stimulates electroneutral NaCl absorption but not electrogenic Na^+^ absorption (5). The question therefore arises as to whether the presence of apical Na^+^ channels (as part of the electrogenic Na^+^ absorptive process induced by aldosterone in rat distal colon during dietary K^+^ loading) leads to depolarization of the apical membrane, which might be expected to promote BK channel-mediated apical K^+^ exit. Indeed, intracellular microelectrode studies in rat distal colon indicated that apical Na^+^ and K^+^ channels coexist in surface cells and upper crypt cells during increased colonic K^+^ secretion induced by dietary Na^+^ depletion and dietary K^+^ loading (22, 31). However, electrogenic K^+^ secretion induced in rat distal colon by dietary Na^+^ depletion was unchanged after the inhibition of electrogenic Na^+^ absorption by amiloride (6), and aldosterone added to guinea pig distal colon in vitro stimulated an active K^+^ secretory process that was independent of Na^+^ absorption (28). Furthermore, microelectrode studies have identified significant TEA-inhibitable apical (K^+^) conductances in the distal colon of both dietary Na^+^-depleted and dietary K^+^-loaded rats following apical Na^+^ channel inhibition by luminal amiloride (22, 31), which suggest that hyperpolarization of the apical membrane in response to Na^+^ channel inhibition had little or no effect on apical BK channel activity, and thus on K^+^ secretion. On the other hand, secondary hyperaldosteronism does not induce apical Na^+^ channels in rat proximal colon (5), and the fact that the overall K^+^ secretory response in this colonic segment is smaller than that in the distal segment (8, 9, 15) may solely reflect the stimulatory effect of Na^+^-H^+^ exchange-mediated cytosolic alkalinization on BK channel activity.

#### Distribution of mineralocorticoid receptors in rat colon.

As a final point, the previously mentioned qualitative difference in aldosterone-induced Na^+^ transport between rat proximal and distal colon during dietary Na^+^ depletion and, as a corollary, the segmental difference in apical BK channel expression during dietary K^+^ loading, may reflect regional variability in aldosterone binding to mineralocorticoid receptors. Indeed, in rat colon, the number of mineralocorticoid receptor binding sites is substantially greater in the distal segment than in the proximal segment (41). Thus the ability of chronic K^+^ loading to increase apical BK channel expression in the distal colon, but not in the proximal colon, may simply reflect a segmental difference in mineralocorticoid receptor activation in response to the relatively modest level of secondary hyperaldosteronism (24). It remains to be established whether the much higher level of secondary hyperaldosteronism induced by dietary Na^+^ depletion results in similar increases in apical BK channel expression in rat proximal and distal colon. If that were not to be the case, it would suggest that the segmental difference in apical BK channel expression during chronic K^+^ loading reflects an inherent difference in response between the two target epithelia, which is not dependent on the number of mineralocorticoid receptors.

#### Summary.

We have shown that K^+^ adaptation in rat colon induced by chronic dietary K^+^ loading involves greater levels of apical BK channel expression and activity in the distal segment than in the proximal segment. Chronic K^+^ loading also results in greater amplification of basolateral membrane area and Na^+^-K^+^-ATPase-mediated basolateral K^+^ uptake in the distal colon than in the proximal colon (19). Taken together, these adaptive changes likely underlie the greater net K^+^ secretory response that occurs in the distal colon compared with the proximal colon during chronic dietary K^+^ loading (8, 9, 15). We have also shown apical BK channel expression along the surface cell-crypt cell axis to be increased in patients with end-stage renal disease (25), in whom renal K^+^ excretion is markedly decreased, while the capacity of the colon for K^+^ secretion is increased (32, 33). Thus upregulation of colonic apical BK channel expression, leading to an enhanced luminal K^+^ conductance, is likely to be an important factor in transforming the colon into a major accessory organ of K^+^ homeostasis in these patients (25). We conclude that upregulation of apical BK channels is an important part of the adaptive mechanisms ensuring increased colonic K^+^ secretion in response to dietary K^+^ enrichment and decreased renal K^+^ excretion.

## GRANTS

This work was supported by a Wellcome Trust project grant and the West Riding Medical Research Trust (G. I. Sandle) and NIH/NIDDK DK-018777 project grant (V.M. Rajendran).

## DISCLOSURES

No conflicts of interest, financial or otherwise, are declared by the author(s).

## AUTHOR CONTRIBUTIONS

M.D.P., V.M.R., and K.M. performed experiments; M.D.P., V.M.R., K.M., and G.I.S. analyzed data; M.D.P., V.M.R., K.M., and G.I.S. interpreted results of experiments; M.D.P., V.M.R., K.M., and G.I.S. approved final version of manuscript; K.M. and G.I.S. prepared figures; G.I.S. conception and design of research; G.I.S. drafted manuscript; G.I.S. edited and revised manuscript.
